# Diet variability among insular populations of *Podarcis* lizards reveals diverse strategies to face resource‐limited environments

**DOI:** 10.1002/ece3.5626

**Published:** 2019-09-30

**Authors:** Maxime Taverne, Anne‐Claire Fabre, Nina King‐Gillies, Maria Krajnović, Duje Lisičić, Louise Martin, Leslie Michal, Donat Petricioli, Anamaria Štambuk, Zoran Tadić, Chloé Vigliotti, Beck A. Wehrle, Anthony Herrel

**Affiliations:** ^1^ UMR 7179 Département Adaptations du Vivant Muséum National d'Histoire Naturelle Centre National de la Recherche Scientifique Paris France; ^2^ Life Sciences The Natural History Museum London UK; ^3^ Department of Biology Faculty of Science University of Zagreb Zagreb Croatia; ^4^ D.I.I.V. Ltd. for Marine, Freshwater and Subterranean Ecology Sali Croatia; ^5^ Department of Ecology & Evolutionary Biology University of California Irvine CA USA

**Keywords:** Croatia, diet, insularity, lizard, *Podarcis*, sexual dimorphism

## Abstract

Access to resources is a dynamic and multicausal process that determines the success and survival of a population. It is therefore often challenging to disentangle the factors affecting ecological traits like diet. Insular habitats provide a good opportunity to study how variation in diet originates, in particular in populations of mesopredators such as lizards. Indeed, high levels of population density associated with low food abundance and low predation are selection pressures typically observed on islands. In the present study, the diet of eighteen insular populations of two closely related species of lacertid lizards (*Podarcis sicula* and *Podarcis melisellensis*) was assessed. Our results reveal that despite dietary variability among populations, diet taxonomic diversity is not impacted by island area. In contrast, however, diet disparity metrics, based on the variability in the physical (hardness) and behavioral (evasiveness) properties of ingested food items, are correlated with island size. These findings suggest that an increase in intraspecific competition for access to resources may induce shifts in functional components of the diet. Additionally, the two species differed in the relation between diet disparity and island area suggesting that different strategies exist to deal with low food abundance in these two species. Finally, sexual dimorphism in diet and head dimensions is not greater on smaller islands, in contrast to our predictions.

## Graphical abstract

Our manuscript investigates the dietary adaptations in ecologically contrasted insular populations of Podarcis lizards. We quantitatively tested whether the resource use in these animals was impacted by the ecological conditions encountered by lizards in island communities, specifically low food abundance and high population density. For this purpose, we conducted a comparative study across eight populations of *Podarcis sicula* and 12 populations of *Podarcis melisellensis* to explore relationship between island size and taxonomic diversity, disparity, and functional properties of the food items found in the stomachs of the animals.

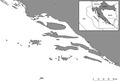

## 1 INTRODUCTION

Access to resources in ecosystems and communities is a dynamic and multicausal process that determines the success and survival of a population. The mechanisms underlying the flux of resources between species sharing the same environment have been investigated in some detail (Lindeman, 1942; Petchey, Beckerman, Riede, & Warren, 2008; Sousa, Domingos, & Kooijman, 2008; Yvon‐Durocher & Allen, 2012) and many factors have been shown to influence dietary variation in vertebrate communities. On one hand, biotic factors including intraspecific and interspecific interactions such as competition between closely related species (Pacala & Roughgarden, 1982), predation (Hawlena & Pérez‐Mellado, 2009; Szarski, 1962), or male–male combat and territory defense (Edsman, 1986) may impact the resources available to an animal. On the other hand, abiotic constraints such as climate or season will influence food availability and thus constrain what is available for an animal to eat (Korslund & Steen, 2006; Renton, 2001). Because of the complexity and the multiplicity of the factors affecting diet, it is often difficult to disentangle these effects.

Whereas mainland ecosystems are highly complex, islands may provide simpler ecosystems where the factors that drive variation in diet can be more easily understood (Kueffer, Drake, & Fernandez‐Palacios, 2014; Losos & Ricklefs, 2009). First, immigration and emigration are limited on islands. Second, islands often host species‐poor communities compared to similar ecosystems on the mainland as a result of a lower ratio between speciation and extinction (Losos & Ricklefs, 2009; Losos & Schluter, 2000). Smaller islands also often lack top predators (Grant, 1972; Losos & Queiroz, 1997), especially carnivorous mammals (MacArthur & Wilson, 1967). As a consequence, predation pressure and interspecific competition are often reduced on islands (Thomas, Meiri, & Phillimore, 2009). This may induce ecological release (Buckley & Jetz, 2007), also called niche expansion, a phenomenon where a population takes advantage of the low intensity of predation pressure to thrive and to broaden their niche width. Mesopredators in particular have been demonstrated to show this phenomenon (Litvaitis & Villafuerte, 1996; Ritchie & Johnson, 2009). Consequences of the ecological release, also known as the island syndrome, impact many aspects of the organisms' ecology, such as aggressiveness, body size, life expectancy, and dietary habits (Adler & Levins, 1994; Meiri, Dayan, & Simberloff, 2006).

Ecological release often drives an increase in population density in insular communities (Buckley & Jetz, 2010; Case, 1975; Hasegawa, 1994) through density compensation. Although not all organisms show this, most vertebrate taxa do, especially lizards (Case, 1975; Pérez‐Mellado et al., 2008; Schoener, 1989) which have been shown to drastically increase their population density when the diversity in species within the community decreases. In this sense, insular populations diverge from their mainland relatives by displaying a higher ratio between intraspecific and interspecific competition (Itescu, Schwarz, Meiri, & Pafilis, 2017; Pafilis, Meiri, Foufopoulos, & Valakos, 2009).

However, prey diversity also drops dramatically on islands. Arthropods, an important food resource for mesopredators such as lizards, do not compensate low diversity by increasing population density, which leads to a collapse of prey biomass on islands (Olesen & Jordano, 2002; Olesen & Valido, 2003). Consequently, the typical food resources may become too scarce to provide the needs of an increased population of mesopredators, resulting in dietary changes and the inclusion of new food sources (e.g., less nutritional items such as plants) (Bolnick, 2001; Van Damme, 1999). Therefore, high population densities combined with low food abundance will result in intense competition for access to food which may subsequently drive dietary variation in insular populations.

Previous studies have indeed suggested that intraspecific competition for resource access can increase variation in diet within insular populations (Svanbäck & Bolnick, 2007). This is likely the case because ecological release promotes morphological variation enabling animals to exploit novel food resources (Thomas et al., 2009). The intrapopulation diversification of diet subsequent to the invasion of new habitats with low food abundance was suggested to be advantageous by reducing diet overlap and increase food partitioning. Therefore, intense intraspecific competition associated with food scarcity may drive dietary adaptations. Examples of diet diversification in such populations are common, for example in birds (MacArthur, Diamond, & Karr, 1972) and lizards (at the interspecific level, see Schoener, Slade, & Stinson, 1982; Herrel, Vanhooydonck, & Van Damme, 2004; at the intraspecific level, see Schoener, 1967; Brown & Pérez‐Mellado, 1994; Sagonas et al., 2014; Donihue, Brock, Foufopoulos, & Herrel, 2016).

The present study aims to characterize the diversity of diet across insular populations of two closely related species of lacertid lizards: seven populations of *Podarcis sicula* (Rafinesque‐Schmaltz, 1810) and eleven of *Podarcis melisellensis* (Braun, 1877). The populations of interest provide a unique opportunity to unravel the factors affecting dietary diversity as they live in ecologically relatively simple insular systems. They often share their habitat with few other lizard species, but most frequently with the rock specialist *Dalmatolacerta oxycephala* and the nocturnal gecko *Hemidactylus turcicus*. Consequently, resource availability and intraspecific competition are likely the main drivers of variation in diet. These lizards were studied on several islands in the middle Adriatic, off the coast of Croatia. These islands further differ in size, yet are within a relatively short distance from one another.

*Podarcis sicula* is known to be more aggressive than its close relative *P. melisellensis*. It is also invasive in many areas worldwide suggesting it is a more generalist species. The way both species deal with the variable intensity of intraspecific competition may thus differ and might have an impact on dietary variation. Moreover, sexual dimorphism, especially in body size, is important in lizards living on small and depauperate islands (Schoener, 1977) and has been suggested as determinant factor in food partitioning between males and females (Perry, 1996). Finally, the populations included in the present study live on islands of very diverse size, structure, and vegetation cover that all may impact population density and food availability (Polis & Hurd, 1996). We formulate four hypotheses that will be tested in this study: (a) we expect to find significant differences between species, sexes, and islands; (b) we expect that the diversity in diet is correlated with island size since food diversity and abundance are dependent on island size; (c) we expect the populations from the smallest islands to present greater levels of sexual dimorphism as a response to increased intraspecific competition for food resources; and (d) we expect to find a lower disparity in the diet of the populations of *P. melisellensis* compared to populations of the more generalist *P. sicula*.

## 2 MATERIALS AND METHODS

### 2.1 Specimens

**Figure 1:**
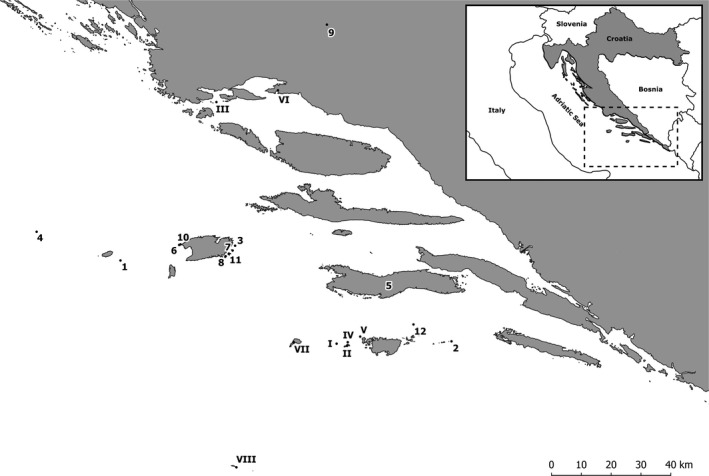
Location of the populations of *Podarcis sicula* (I: Bijelac; II: Kopište; III: Pijavica; IV: Pod Kopište; V: Pod Mrčaru; VI: Split; VII: Sušac; VIII: Mala Palagruža) and *Podarcis melisellensis* (1: Brusnik; 2: Glavat; 3: Grebeni; 4: Jabuka; 5: Korčula; 6: Mali Barjak; 7: Mali Paržanj; 8: Ravnik; 9: Sinj; 10: Veli Barjak; 11: Veli Budikovac; 12: Veli Tajan) sampled for the present study

Following the issuance of the permit from the Ministry of the Environmental Protection of the Republic of Croatia, Directorate for Nature Protection, specimens of *P. sicula* were captured across eight sites (including one continental site) and *P. melisellensis* across twelve sites (including one continental site) (Figure 1). The two species were never found to coexist on the islands. In total, 535 adult animals were captured by noose or by hand at the end of the summer 2016 (Table 1). The snout–vent length (SVL), and linear head dimensions such as head length (HL), head width (HW), head height (HH), lower jaw length (LJL), quadrate‐to‐tip length (QT), and coronoid‐to‐tip length (CT) of all individuals were measured using digital calipers (Mitutoyo absolute digimatic; ±0.01 mm).

**Table 1:** List of the specimens and their origin

Species	Site	Symbol	Area (m^2^)	Date	Females	Males
*Podarcis melissellensis*	Brusnik	BR	13,791	08.27.2016	22	20
	Glavat	GL	3,379	09.09.2016	9	12
	Grebeni	GR	9,187	08.31.2016	12	18
	Jabuka	J	22,585	08.28.2016	21	17
	Korčula	KR	2.79 × 10^8^	10.08.2016	7	11
	Mali Barjak	BM	3,632	08.26.2016	14	18
	Mali Paržanj	PZ	2,671	09.01.2016	13	12
	Ravnik	RV	55,140	09.03.2016	6	11
	Sinj^a^	SI		09.12.2016	5	11
	Veli Barjak	BU	1,246	08.30.2016	19	21
	Veli Budikovac	BD	63,852	09.02.2016	17	17
	Veli Tajan	T	2,702	09.08.2016	7	8
*Podarcis sicula*	Bijelac	BI	734	09.08.2016	7	13
	Kopište	KO	155,049	09.06.2016	14	19
	Mala Palagruža	PG	5,036	09.10.2016	8	12
	Pijavica	PI	2,059	09.14.2016	13	12
	Pod Kopište	K	7,915	09.06.2016	15	22
	Pod Mrčaru	M	2,931	09.05.2016	12	24
	Split^a^	ST		09.12.2016	9	7
	Sušac	SU	853,927	09.04.2016	15	5

a Continental sites.

### 2.2 Island area

The area of each island was determined using Google Earth.

### 2.3 Stomach contents analysis

All individuals were stomach flushed immediately after capture using a syringe with water and a ball‐tipped steel needle (Herrel, Joachim, Vanhooydonck, & Irschick, 2006; Measey, Rebelo, Herrel, Vanhooydonck, & Tolley, 2011). The animals were tapped gently on the sides of the jaw, resulting in a typical gaping response. A small plastic ring was then inserted between the jaws allowing the introduction of the needle into the pharynx. Palpation helped to detect the position of the needle and to further insert it through the digestive tract into the stomach. The water was then slowly squeezed out of the syringe, and food was pushed out with the water. We maintained the flow of water until only water flowed out of the stomach. The stomach contents were preserved in individual vials containing a 70% aqueous ethanol solution and labeled. Animals were measured and marked using a nontoxic marker to distinguish them and to make sure that they were manipulated only once. A recovering time of 12 hr was then observed before releasing the animals to their exact site of capture.

### 2.4 Characterization of the diet

The prey items of each stomach content were identified down to the lowest possible taxonomic order following Chinery (1986), and counted. Each food item (including plant matter) was weighted using a Mettler electronic balance (±0.1 mg), and a dial caliper (Mitutoyo ± 0.2 mm) was used to determine their length and width. The volume of each prey item was calculated following a spheroid equation:

$$\text{Volume}=\frac{4}{3}\times \pi \times \frac{L}{2}\times {\left(\frac{W}{2}\right)}^{2}$$

where *L* is the length of the item and *W* its width. However, prey items are not always intact preventing the estimation of their volume. To account for this, we measured the length of several key body parts (i.e., abdomen, head, or wing) and the total length and the width of approximately 15 intact prey items per taxa. For each taxon, these measurements were used to establish a reference equation that further allowed us to estimate the length and the width, and subsequently the total volume, of the damaged prey when a key body part was found in the stomach contents.

Prey was classified according to their functional properties in terms of hardness and evasiveness (Table 2), following Vanhooydonck, Herrel, Van, and Damme (2007). Hardness included three categories (soft, medium, and hard) as has been previously established by quantifying the forces needed to crush a prey item (Aguirre, Herrel, Damme, & Matthysen, 2003; Andrews & Bertram, 1997; Herrel, Spithoven, Damme, & Vree, 1999; Herrel, Damme, Vanhooydonck, & Vree, 2001; Herrel, Vand Damme, & De Vree, 1996; Herrel, Verstappen, & De Vree, 1999). The criterion of evasiveness referred to the ability of the prey to escape a predator before capture and also includes three categories (sedentary, intermediate, and evasive). Plant matter was not included in these calculations and was considered separately. Thus, total of seven food categories was considered in the analyses.

**Table 2:** Functional categories of prey taxa identified in the stomach contents

Hardness	Evasiveness	Prey taxa
Soft	Sedentary	Acari
		Aphidoidea
		Araneae
		(Insecta) Larvae
		Pseudoscorpionida
		Thysanura
	Intermediate	Chilopoda
		Embioptera
		Heteroptera
	Evasive	Diptera
		Lepidoptera
Medium	Sedentary	Diplopoda
		Isopoda
	Evasive	Orthoptera
Hard	Sedentary	Gastropoda
		Homoptera
	Intermediate	Coleoptera
		Formicidae
	Evasive	Flying Hymenoptera

The relative numerical (*N*%) and mass (*M*%) abundance were calculated for each functional food category for each individual, along with the relative contribution of each food category to the whole stomach content. An index of relative importance (IRI) was then calculated following the equation:

$$\text{IRI}=\left(N\%+M\%\right)\times O\%$$

where *O*% is the frequency of occurrence of each prey category (in other words, the proportion of stomachs containing at least one item of the designated food category; Martin, Twigg, & Robinson, 1996; Pinkas, 1971; Twigg, How, Hatherly, & Dell, 1996). Since it is not possible to calculate means for the frequency data, the IRI was determined for each prey category, for each sex, and for each site. Based on all measured and calculated variables, four diet subdatasets are retained and considered separately for the statistical analyses: the first three describe the numerical and mass abundance, and IRI for each of the seven food categories, and the last subdataset includes five prey dimensions. These are *L*_max_ (the maximum length of any prey item ingested by an individual), *L*_min_ (the minimum length), *W*_max_ (the maximum width), *W*_min_ (the minimum width), and *V*_max_ (the maximum volume of any prey item ingested by an individual).

### 2.5 Statistical analyses

All statistical analyses were carried out using R (v3.5.1, R Core Team, 2018). The relative numerical and mass abundance data, which values ranged between 0 and 1, were arcsin‐transformed to normality. Values of prey dimensions and of head dimensions were log_10_‐transformed. Prior to any analysis, the normality and the homogeneity of variances in each subdataset were tested by a Shapiro test and a Bartlett's test.

### 2.6 Variability in diet

A three‐way MANOVA was performed on the whole diet dataset with sex, species, and site as factors as well as for a possible interaction between these factors to test for their effect on diet. Next, MANOVAs were computed for each sex and species separately, with the site as factor (function “MANOVA” in the package “stats”). Tukey's post hoc tests (function “TukeyHSD” in the package “stats”) were used to explore which islands differed from one another.

### 2.7 Diversity and disparity

The taxonomical diversity of prey was determined through the calculation of the Shannon–Wiener's diversity index (*H*′). In the following equation,

$$H^{\prime }=-\sum_{i=1}^{S}p_{i}\log\left(p_{i}\right)$$

“*S*” is the total number of prey taxa found in an individual stomach, and “*p_i_*” is the relative abundance of the prey taxon, calculated as follows:

$$p_{i}=\frac{n_{i}}{N}$$

where *N* is the total number of prey items found in a stomach and *n_i_* the total number of prey items of the taxon *i*. As it is commonly used in ecological studies, we used the logarithm base 2 of this metric for analysis. Because the distribution of *H*′ was found to be bimodal, the difference in sex, species, and island was investigated with Mann–Whitney–Wilcoxon tests (“wilcox.test” function in “stats” package). A possible relationship between diversity in diet and island area or the proportion of plants in the diet was then tested by means of Spearman rank correlation tests (function “cor.test” from the “stats” package). For the latter analysis, the index was averaged by population or subpopulation to avoid pseudo replication.

The disparity index calculates the hypervolume occupied by a subset in ecological space. Based on the two numerical and mass abundance datasets, it was computed using the function “disparity.per.group” from the “dispRity” package (Guillerme, 2018) in R. The comparison of the disparity of subsets allowed us to test for differences between species, sexes, and islands. This was done using the “test.disparity” function which uses a Mann–Whitney–Wilcoxon test of comparison of the medians, coupled with a Bonferroni correction multiple comparisons when required. We also tested for a relationship between disparity and island size. To do so, the disparity metric “sum” was extracted for each site, sex, and species, log_10_‐transformed and regressed against island area. All linear regressions involving island area exclude mainland sites and the island Korčula. Whereas continental sites have no area associated, Korčula is a very large island close to the mainland and thus its community is expected to behave similar to a mainland one.

### 2.8 Sexual dimorphism in diet, prey size, head dimensions and SVL

For both species separately, principal component analyses (PCA) were performed on the datasets describing numeric and volumetric prey consumption, prey dimensions, and head dimensions including all individuals using the function “prcomp” from the “stats” package. This allowed for a reduction in dimensionality. The contribution of each specimen along the three first principal components (PC) was extracted and used to calculate the mean contribution of each sex of each population on these axes. The sexual dimorphism (SD) for each site was determined as follows:

$$\text{SD}=\sqrt{{\left(m_{1}-f_{1}\right)}^{2}+{\left(m_{2}-f_{2}\right)}^{2}+{\left(m_{3}-f_{3}\right)}^{2}}$$

where *m_i_* and *f_i_* refer respectively to the mean contribution of the males and the females of the population of interest along with the PC*_i_*.

This method allowed us to calculate three measures of sexual dimorphism: SD_d_ (diet), SD_p_ (prey size), and SD_h_ (head dimensions). Two other sexual dimorphisms, SD_svl_ (body size) and SD_disp_ (disparity), were obtained as the difference in these variables between males and females. We tested for differences between species in each SD, then for a possible relationship between each SD, using Mann–Whitney–Wilcoxon tests, and between SD and island area with a nonparametric Spearman rank correlation test.

## 3 RESULTS

### 3.1 Variability in diet

The calculation of the IRI scores provides a qualitative overview of the trends in diet variability across islands, species, and sexes (Table 3). In the majority of the populations of both species, the two most representative prey categories are hard and intermediate evasive prey. However, plant matter is less common in the diet of *P. melisellensis* compared to *P. sicula*. For example, on Sušac plant matter represents 76% of the food eaten by *P. sicula* (Table 3).

**Table 3:** IRI scores of the seven food categories (Sedent: sedentary preys, Inter: prey of intermediate evasiveness, f: females, m: males). The highest IRI for each prey functional criteria in each population is marked with a *. Scores of plant matter higher than at least one of the two previously marked scores of the same population are also indicated by *

Species	Island	Sex	Hardness			Evasiveness			Plant
			Soft	Medium	Hard	Sedent.	Inter.	Evasive	
*P. melisellensis*	BR	m	597	38	1,796*	103	2,889*	149	55
		f	931	10	2,984*	649	2,881*	125	12
	GL	m	1,059	56	4,101*	1,636*	785	951	153
		f	1,260	5	3,438*	1,727*	1,207	365	60
	GR	m	999*	84	704	205	797	971*	19
		f	1,098	0	1,172*	334	689	872*	22
	JA	m	1,863*	111	717	733	937*	935	156
		f	1,548*	183	515	1,049*	569	585	357
	KR	m	789	1,237	3,727*	1,743	828	1,886*	0
		f	1,128	252	4,323*	2,795*	720	769	0
	BM	m	1,145*	624	243	628	1,925*	80	9
		f	592	207	885*	729	1,177*	29	109
	PZ	m	823*	155	265	307	2,000*	0	62
		f	2,685*	17	1,461	870	2,711*	591	4
	RV	m	797	5	1,886*	221	1,554*	596	28
		f	351	0	1,428*	211	520*	413	458
	SI	m	268	495	2,294*	241	773	1,432*	63
		f	471	331	2,264*	351	690	1,694*	11
	BU	m	827	0	3,405*	418	3,929*	43	31
		f	969	2	2,921*	953	3,108*	0	67
	BD	m	361	450	1,068*	235	1,025*	546	222
		f	633	41	1,341*	119	1,823*	356	6
	VT	m	296	1,055	1,930*	1,800*	981	39	275
		f	936*	572	628	1,098	1,167*	107	352
*P. sicula*	BI	m	2,043*	445	1,098	1,587	394	1,950*	298
		f	1,410*	50	1,076	727	219	1,992*	86
	KO	m	301	573	1,037*	565	1,001*	154	1,296*
		f	456	78	1,671*	432	1,211*	343	1,217*
	PI	m	741	131	1,162*	485	892*	627	1,411*
		f	642*	97	610	544*	387	374	1,678*
	PK	m	876	24	1,150*	297	2,178*	150	916
		f	703*	59	595	331	943*	234	2,248*
	PM	m	126	216	368*	178	248*	210	1,398*
		f	121	82	530*	192	260*	134	2,565*
	ST	m	549	2,452*	1,186	1,294*	1,010	952	508
		f	516	165	2,661*	870	1,113*	532	352
	SU	m	7	0	703*	8	703*	0	4,403*
		f	133	29	503*	68	786*	21	4,804*
	PG	m	865*	586	130	312	130	1,767*	58
		f	981	1,826*	82	1,246*	82	1,138	0

### 3.2 Quantification of the diet variability

The three‐way MANOVA performed on the whole dataset reveals a strong species (*F*_14,482_ = 17.69, *p* < .001), sex (*F*_14,482_ = 2.32, *p* = .004), and site (*F*_252,6930_ = 2.57, *p* < .001) effect on diet composition. However, no interaction is detected between these three factors (*F*_266,6930_ = 1.01, *p* = .411).

When considering the dataset describing the numerical abundance of the seven food categories, the diet of both species is significantly different (*F*_7,527_ = 17.44, *p* < .001). Although males and females did not differ regarding this dataset (*P. melisellensis*: *F*_1,320_ = 1.47, *p* = .176; *P. sicula*: *F*_1,205_ = 0.84, *p* = .551), the results of the three‐way MANOVA justify to carrying out further analyses by sex. For both species, strong differences are detected between islands (*P. melisellensis*: females: *F*_77,980_ = 1.61, *p* < .001, males: *F*_77,1,148_ = 2.29, *p* < .001; *P. sicula*: females: *F*_49,585_ = 1.98, *p* < .001, males: *F*_49,742_ = 2.81, *p* < .001). If we consider the variation in diet by sex and species separately, variables that globally appear to drive the differences among islands are the proportion of plants, the proportion of soft and medium hard prey and the proportion of evasive prey. The dietary proportions of hard prey do not discriminate islands, except in males of *P. melisellensis* (*F*_11,164_ = 2.02, *p* = .029). The analyses on the mass proportion data show similar results, differences between islands being strongly driven by the volumetric proportion of plants, soft, medium, and evasive prey, and poorly influenced by hard prey, except again in males of *P. melisellensis* (*F*_11,164_ = 2.11, *p* = .016).

### 3.3 Diet diversity and disparity

The Mann–Whitney–Wilcoxon test performed on the diversity index (*H*′) shows a significant signal of species (*p* < .001) and island (*p* < .001), but not sex (*p* = .141). *Podarcis melisellensis* consumes, on average, a greater diversity of prey than *P. sicula* (*H*′_m_ = −0.12, *H*′_s_ = −0.31). The taxonomic diversity of prey ingested does not correlate with the island area, neither when both species are considered together (*p* = .065, *ρ* = −0.461), nor when species are considered separately (*P. melisellensis*: *p* = .132, *ρ* = −0.515; *P. sicula*: *p* = .139, *ρ* = −0.642). Interestingly, a strong negative correlation exists between *H*′ and the proportion of plants included in the diet (*p* < .001, *ρ* = −0.181). This, however, only holds for *P. sicula* (*p* < .001, *ρ* = −0.369; *P. melisellensis*: *p* = .357, *ρ* = 0.053).

**Figure 2:**
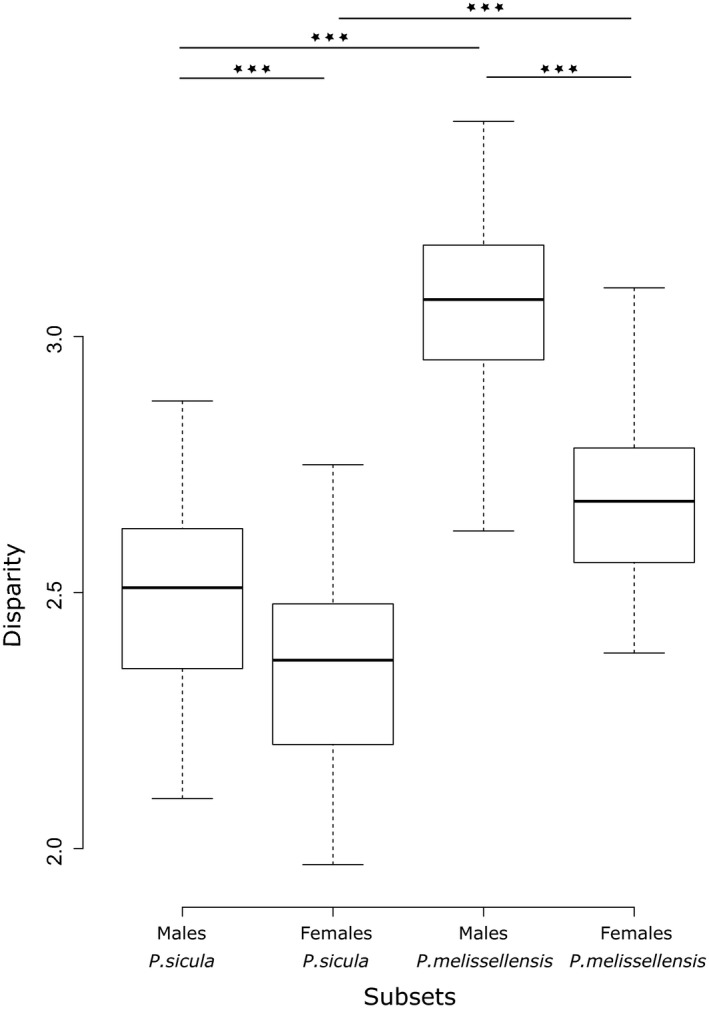
Results of the comparison of the disparity magnitude between species and sexes (middle bars represent median, boxes represent the standard deviation, and the wiskers the minimum and maximum values)

**Figure 3:**
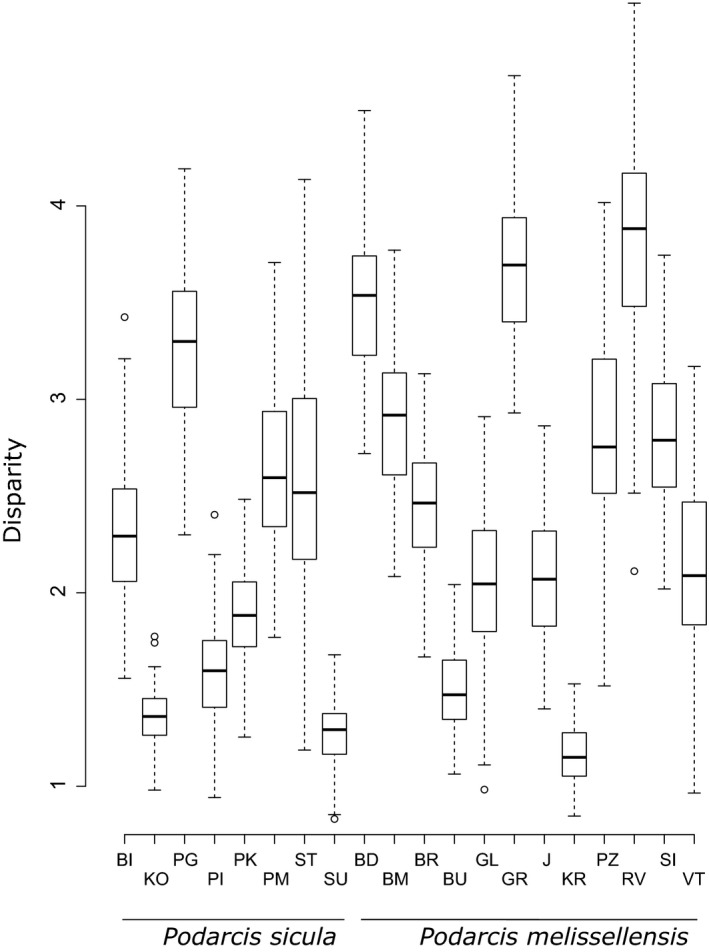
Results of the comparison of the disparity magnitude between populations (middle bars represent median, boxes values represent the standard deviation, and the wiskers the minimum and maximum values)

Analyses performed on the numerical and the mass proportion datasets reveal that the disparity in diet differs between species and sexes (Figure 2). Indeed, the disparity is greater in *P. melisellensis* than in *P. sicula* (*p* < .001), and greater in males than in females (*p* < .001). This difference remains statistically significant, even after a Bonferroni correction, and when each sex of each species is considered separately (*p* < .001) with males of *P. sicula* having lower disparity than females of *P. melisellensis* (Table 4). Moreover, the difference in diet disparity between sexes (SD_disp_) in *P. melisellensis* is greater than in *P. sicula* (*p* < .001). Our results also show a great variability among islands (Figure 3). The lizards with the lowest disparity in diet are those from Kopište, Sušac, Veli Barjak and Korčula, whereas Mala Palagruža, Veli Budikovac, Grebeni and Ravnik have the highest disparity. The disparity magnitude, estimated by the metric “sum” from the “dispRity” package, correlates positively with the island area for females of *P. melisellensis* (*p* = .011, *R* ^2^ = 0.516, intercept = −0.546, slope = 0.246) and negatively for females of *P. sicula* (*p* = .039, *R* ^2^ = 0.527, intercept = 0.755, slope = −0.110).

**Table 4:** Results of disparity analyses: *n*, the number of specimens of each subset; obs, the observed disparity; median, the median value of disparity; and four different percentiles. Results of Wilcoxon tests between each subset, the associated *W*‐values and *p*‐values (s: sicula, m: melisellensis)

Subsets	*n*	Obs	Median	2.5%	25%	75%	97.5%
*sicula*	207	2.430	2.395	2.101	2.301	2.472	2.687
*melisellensis*	328	2.917	2.883	2.659	2.819	2.968	3.125
Males	290	3.004	2.968	2.759	2.897	3.052	3.179
Females	245	2.727	2.705	2.435	2.623	2.778	2.937
Males *sicula*	114	2.506	2.509	2.161	2.353	2.625	2.815
Females *sicula*	93	2.348	2.368	1.984	2.204	2.475	2.703
Males *melisellensis*	176	3.093	3.072	2.705	2.955	3.175	3.363
Females *melisellensis*	152	2.704	2.678	2.447	2.560	2.782	3.042

Test	*W*	*p*‐Value					
*sicula*/*melisellensis*	54	<0.001*					
Males/females	541	<0.001*					
Males *s*/females *s*	6,986	<0.001*					
Males *s*/males *m*	126	<0.001*					
Males *s*/females *m*	2,338	<0.001*					
Females *s*/males *m*	51	<0.001*					
females *s*/females *m*	882	<0.001*					
Males *m*/females *m*	9,377	<0.001*					

* Significant *p*‐values.

### 3.4 Sexual dimorphism

Spearman rank tests did not reveal any significant relationships between island area, the degree of sexual dimorphism in morphology, or diet (Table 5). Mann–Whitney–Wilcoxon tests did not show any difference in the sexual dimorphism between the two species except in head dimensions, for which sexual dimorphism is greater in *P. melisellensis* than in *P. sicula* (*p* = .006, mean_s_ = −0.746, mean_m_ = −0.604).

**Table 5:** Results of the Spearman tests for rank correlation between the different sexual dimorphisms (SD_d_: in diet, SD_p_: in prey dimensions, SD_svl_: in body size, SD_h_: in head dimensions, SD_disp_: in diet disparity) and island area. The table gives the *ρ*‐value and the *p*‐value of each test. Pairs marked with an “x” were not tested

	Variable	*P. melisellensis*						
		Area	SD_d_	SD_p_	SD_svl_	SD_h_	SD_disp_	
*P. sicula*	Area		0.236	0.321	0.321	0.393	−0.624	*ρ*
			0.514	0.367	0.367	0.263	0.060	*p*
	SD_d_	−0.071		x	0.200	0.357	x	*ρ*
		0.906		x	0.583	0.313	x	*p*
	SD_p_	−0.035	x		0.345	0.478	x	*p*
		0.963	x		0.331	0.166	x	*p*
	SD_svl_	−0.107	−0.428	0.536		x	−0.163	*ρ*
		0.839	0.356	0.236		x	0.657	*p*
	SD_h_	0.178	−0.071	0.392	x		−0.163	*ρ*
		0.713	0.906	0.396	x		0.657	*p*
	SD_disp_	0.428	x	x	0.178	0.250		*ρ*
		0.353	x	x	0.713	0.594		*p*

## 4 DISCUSSION

The present study highlights the inter‐ and intraspecific variability in dietary resources consumed by two closely related species of *Podarcis* lizards. The lack of an interaction between the factors of the general three‐way MANOVA (species, sex, and island) suggests that variation in diet differs by island irrespective of the sex and species considered. Similarly, differences between sexes are independent of variation between species and islands, and finally variation between species is not dependent on the island nor the sex of the individual.

### 4.1 Among island variability in dietary diversity

Insularity profoundly affects the dynamics of a population. Intraspecific competition, enhanced in insular habitats compared to similar ecosystems on mainland, is known to promote dietary diversification (Svanbäck & Bolnick, 2007). We hypothesized that intraspecific competition would increase in parallel to population density when island size decreases (Donihue et al., 2016). We then predicted that taxonomic diversity in diet would be inversely proportional to island size. Interestingly, our results do not show any relationship between island size and dietary diversity. Two hypotheses can be formulated to explain this result. First, the diversity of prey available can be so high that its variation, impacted by island area, does not affect diversity in diet. Second, below a certain diversity threshold of available prey, lizards do not change their dietary width but a shift in the diet resources exploited does occur, resulting in an apparent stasis in the diversity in diet. These hypotheses are supported by the fact that most lacertid lizards are known to be mostly food generalists (Arnold, 1987; Diaz, 1995) whose diet globally corresponds to the available resources (Mou & Barbault, 1986; Pollo & Pérez‐Mellado, 1988). It has been previously suggested that food generalists would rather feed on a constant prey diversity while specialists would take a greater variety of prey when available resource diversity drops (Olsen, Fuentes, Bird, Rose, & Judge, 2008), which is congruent with our results. Even if food availability, and consequently diet, fluctuate over the year, diet diversity in lacertid lizards tends to remain constant (Diaz & Carrascal, 1993; Pérez‐Mellado et al., 1991). In this case, it seems that the opportunistic feeding habits of lizards are a possible explanation for the lack of relationships between taxonomic prey diversity in the diet and island size. However, an investigation on food availability on these islands is required to test for food electivity in these populations and could shed further light on the observed patterns.

Insular populations of *P. sicula* might also shift to a more omnivorous diet, as is seen on the islands Sušac, Pijavica and Pod Mrčaru, where populations include an important proportion of plant items into their diets. Eating plants requires more foraging time, thus exposing lizards to potential predators (Adamopoulou & Legakis, 2002; Hawlena & Pérez‐Mellado, 2009; Szarski, 1962). On islands, this shift is often thought to be facilitated by a reduction in predation pressure and is enhanced on the smallest islands (Schoener et al., 1982). Plant consumption is highly variable among islands, ranging from strictly insectivorous populations (e.g., Brusnik or Mala Palagruža) to almost completely herbivorous populations (e.g., Sušac or Pod Mrčaru). The large amount of plant material found in stomachs of *P. sicula* lizards (see also Herrel et al., 2008; Vervust, Pafilis, Valakos, Grbac, & Damme, 2010) seems to be enabled by their generalist habits.

### 4.2 Dimorphism and within‐population resource partitioning

The ability to deal with different prey items is often accompanied with changes in head dimensions (Arnold, 1987) providing a mechanical advantage (e.g., bite force, gape) in the processing of items of different size and hardness. Territorial males typically bite harder than females thus enabling them to obtain access to a broader range of food resources (Herrel et al., 2001; Herrel, Verstappen, et al., 1999). The results of the present study confirm our prediction that disparity in diet, which represents the total diversity of items ingested at the population level, is higher in males than in females (although this dietary dimorphism does not hold when considering taxonomic diversity). Nevertheless, an amplification of sexual dimorphism concomitant with a decrease in island size was not confirmed, neither in diet data nor in head dimensions. The feeding apparatus in lizards is influenced by more than just feeding constraints. Sexual selection driving male‐based sexual dimorphism in head size, and consequently bite force, is also considered important in male–male interactions (Huyghe, Vanhooydonck, Scheers, Molina‐Borja, & Damme, 2005; Lailvaux, Herrel, Vanhooydonck, Meyers, & Irschick, 2004). In *Podarcis* lizards particularly, the intensity of sexual dimorphism in head size is often, yet not always, correlated with the intensity of dimorphism in prey size ingested (see Vincent & Herrel, 2007 for a review), suggesting that natural selection may not always be the main factor responsible for the emergence of the head dimorphism. Furthermore, despite a clear difference in head dimensions and diet composition between males and females, the lack of any relationship between sexual dimorphism in head dimensions and dimorphism in diet suggests a central role of sexual selection in the emergence of head size dimorphism in the two species examined here. In summary, our results show that (a) a male‐based sexual dimorphism in head dimensions and diet (disparity, prey dimensions, and prey proportions) exists, (b) that the intensity of dimorphism in head dimensions does not correlate with that in diet, suggesting that it is primarily driven by sexual selection, and (c) that the intensity of dimorphism is not amplified on the smallest islands, suggesting that if increased intraspecific competition is occurring on small islets, this likely results in both sexes occupying different microhabitats.

### 4.3 Interspecific variation in diet

Disparity in the diet of *P. melisellensis* is higher than that in *P. sicula*. This result is counter‐intuitive since it is generally assumed that *P. sicula* is an invasive and more generalist species likely consuming a wide array of prey resources (Zuffi & Giannelli, 2013). Moreover, although diet disparity is positively correlated with the island area in *P. melisellensis*, disparity increases with decreasing area in *P. sicula*. This underlines the ability of the latter to forage on a wider variety of food items when food availability is reduced. Bolnick et al. (2010) described several patterns of ecological release following island invasion. The total niche width of the population (TNW) has two additive components that contribute to its variation: the within‐individual and the between‐individual variance (respectively WIC and BIC). An increase in the total niche width can be due to an increase in within‐individual variance (parallel release) or to an increase in between‐individual variance (niche variation release) (Grant, Grant, Smith, Abbott, & Abbott, 1976; Van Valen, 1965), or both. Despite the fact that our data does not allow us to address these issues precisely we suggest that dietary disparity, being an estimate of the total dietary variance of the population, can be likened to the total niche width. Moreover, Shannon's diversity index may be a relevant substitute for variance (Bolnick, Yang, Fordyce, Davis, & Svanbäck, 2002) and is calculated at the individual level. An index of diversity that remains constant with island size variation would suggest that the within‐individual component remains constant, while the total population niche width and subsequently the between‐individual component, represented by the disparity, would follow the pattern previously described. Following this scheme, it is probable that the populations of *P. sicula* increase their niche width when islands are smaller, and that populations of *P. melisellensis* do the opposite. In both species, this variation in population niche width appears to be driven by the between‐individual component, suggesting the occurrence of individual specialization toward different types of food items (Bolnick et al., 2010). As this only holds for females of both species, it also implies that males and their diet are under the strong influence of sexual selection.

### 4.4 Limits and robustness of the present study

Studying diet presents many difficulties (Carretero, 2004). Diet in lizards is, for example, highly variable between seasons (Gadsen & Palacios‐Orona, 1997; Mamou, Marniche, Amroun, & Herrel, 2016). To prevent seasonal biases, we sampled the stomach contents of all our populations within a 45 day‐long period (19 days when excluding Korčula which was sampled slightly later). This enables a reliable comparison of the diet between sites, yet does not account for possible island‐specific seasonal variation in diet. Intraspecific competition and resource availability are also highly season‐dependent. We sampled at the end of the summer when food availability is lowest in these Mediterranean habitats (Karamaouna, 1987), thus increasing the likelihood of detecting differences between islands if specialization occurs. Moreover, nectarivory has been shown to be quite common across lacertid species, especially among *Podarcis* (Pérez‐Mellado & Traveset, 1999). Although nectar can provide alternative resource opportunities, it cannot be detected in the stomachs and possibly impacts dietary patterns. Isotope analyses of the stomach contents could help determining in what amount the lizards include nectar into their diet. Finally, even if the regressions associated with the patterns described here are statistically significant, they explain only a small part of the overall variance. Yet, it is important to note that despite the huge variability and contingency of diet sampling (individuals, location of sampling, time of the day, sunshine, temperature, state of digestion, etc.) we were able to detect biologically meaningful trends. Diet therefore appears to be a central ecological variable impacted by habitat structure and biotic interactions with conspecifics in these two species of *Podarcis* lizards.

## 5 CONCLUSION

The present study illustrates the diversity in diet across insular populations of two *Podarcis* species within the same geographic area. Significant differences in dietary composition were found between sexes, among populations, and between species. The lack of a relationship between taxonomic diversity in diet and island area may be explained by the opportunistic habits described for these species. In the case of low food abundance conditions, animals might shift their diet toward more omnivorous diet, as is observed in *P. sicula* that includes a significant amount of plant matter in its diet on some islands. The investigation of disparity suggests that both species have different strategies when facing food scarcity on the smallest islands. Indeed, in contrast to females of *P. melisellensis*, females of *P. sicula* seem to be able to widen their food niche to counteract the low prey abundance. Although diet disparity was found to be greater in males than in females, neither this dimorphism nor the dimorphism in diet or head dimensions was amplified on the smaller islands. These two last results suggest that dietary specialization in males is also driven by other important factors such as sexual selection. In this sense, our observations on the dimorphism of head dimensions and diet suggest that the differences between sexes might be explained by the occupation of different microhabitats rather than different food niches.

## CONFLICT OF INTEREST

The authors declare no conflict of interest.

## AUTHOR CONTRIBUTIONS

AH designed the study. ACF, NKG, MK, DL, DP, AS, ZT, CV, BAW, and AH collected the field data. MT, LMa, and LMi identified stomach contents. MT performed statistical analyses. MT drafted the manuscript, and all the authors contributed, read, and approved the final version.

## Data Availability

Raw data are available online on Dryad repository (https://doi.org/10.5061/dryad.2b1g52j).
